# Understanding and applying the RE-AIM framework: Clarifications and resources

**DOI:** 10.1017/cts.2021.789

**Published:** 2021-05-14

**Authors:** Jodi Summers Holtrop, Paul A. Estabrooks, Bridget Gaglio, Samantha M. Harden, Rodger S. Kessler, Diane K. King, Bethany M. Kwan, Marcia G. Ory, Borsika A. Rabin, Rachel C. Shelton, Russell E. Glasgow

**Affiliations:** 1 University of Colorado, School of Medicine, Aurora, CO, USA; 2 University of Nebraska Medical Center, Omaha, NE, USA; 3 Patient-Centered Outcomes Research Institute (PCORI), Washington, DC, USA; 4 Virginia Polytechnic Institute and State University, Blacksburg, VA, USA; 5 Arizona State University, College of Health Solutions, Phoenix, AZ, USA; 6 University of Alaska Anchorage, Center for Behavioral Health Research and Services, Institute of Social and Economic Research, Anchorage, AK, USA; 7 Texas A&M University, College Station, TX, USA; 8 University of California San Diego, La Jolla, CA, USA; 9 Columbia University, Mailman School of Public Health, New York, NY, USA

**Keywords:** RE-AIM, implementation science framework, generalization, PRISM, context, adaptation, sustainability

## Abstract

**Introduction:**

Understanding, categorizing, and using implementation science theories, models, and frameworks is a complex undertaking. The issues involved are even more challenging given the large number of frameworks and that some of them evolve significantly over time. As a consequence, researchers and practitioners may be unintentionally mischaracterizing frameworks or basing actions and conclusions on outdated versions of a framework.

**Methods:**

This paper addresses how the RE-AIM (Reach, Effectiveness, Adoption, Implementation, and Maintenance) framework has been described, summarizes how the model has evolved over time, and identifies and corrects several misconceptions.

**Results:**

We address 13 specific areas where misconceptions have been noted concerning the use of RE-AIM and summarize current guidance on these issues. We also discuss key changes to RE-AIM over the past 20 years, including the evolution to Pragmatic Robust Implementation and Sustainability Model, and provide resources for potential users to guide application of the framework.

**Conclusions:**

RE-AIM and many other theories and frameworks have evolved, been misunderstood, and sometimes been misapplied. To some degree, this is inevitable, but we conclude by suggesting some actions that reviewers, framework developers, and those selecting or applying frameworks can do to prevent or alleviate these problems.

## Introduction

Implementation science benefits from theories, models, or frameworks (referred to as TMFs), which are used to (a) guide decisions, hypothesis generation, measure selection, and analyses; (b) enhance generalizability; and (c) improve outcomes or at least understanding [1,2]. Use of a TMF has become a criterion for funding implementation science proposals. Given their critical role, TMFs should also evolve over time to reflect research findings and newer applications [3]. Keeping pace with TMFs, as well as operationalizing and applying them, can be challenging [4].

Because there are so many TMFs in implementation science – over 150 in one recent review [5] – and because they differ in type, purpose, and uses, it is helpful to have ways to organize, understand, and help researchers select the most appropriate TMFs for a given purpose [1]. Equally important, when researchers select or describe a TMF, they should review the most current writings and resources, rather than rely solely on the seminal paper or a review, since TMFs evolve as they are applied to different contexts and behaviors. For example, the PARIHS framework was modified, updated, and reintroduced as iPARIHS [6]. This issue is important because as Kislov *et al.* have articulated, TMFs need to evolve in response to data, new challenges, and changing context, rather than becoming ossified [3].

Given the complexity of the issues involved and magnitude of the literature on TMFs, researchers frequently – and understandably – refer to foundational papers or reviewer characterizations without searching for more recent articles on a TMF. Early categorizations then get perpetuated through implementation science courses, training programs, textbooks, later studies, review sections, and reviews of reviews. Consequently, researchers may not recognize the evolution, refinement, or expansion of TMFs over time as they are adapted, expanded, empirically tested, or applied in new ways, settings, or populations.

This has occurred with the RE-AIM framework [7,8]. Often reviews of TMFs put firm boundaries around types of TMFs (e.g., as either explanatory, process, or outcome; can be used only for evaluation vs planning vs improvement). In reality, these boundaries are often blurry, and some TMFs may fit in more than one category. RE-AIM concepts and measures of key constructs have evolved since the initial RE-AIM publication, in line with emerging data and advances in research methods and implementation science more broadly. These issues and resulting misconceptions are discussed below.

It is important to make efforts to prevent and reduce confusion concerning the application of TMFs. Explicitly clarifying misconceptions, either because of misunderstanding or evolution of a TMF over time can accelerate the advancement of implementation science. Advancing understanding will reduce unintended consequences such as pigeonholing the application of TMFs, stifling innovation, and negatively impacting investigators.

The purposes of this paper are to (1) summarize key ways that RE-AIM has evolved over time and how this relates to misinterpretations; (2) identify specific misconceptions about RE-AIM and provide corrections; (3) identify resources to clear up confusion around use of RE-AIM; and (4) offer recommendations for TMF developers, reviewers, authors, and users of scientific literature to reduce the frequency of or mitigate the impact of TMF misinterpretations.

### The Evolution of RE-AIM

Many TMFs evolve over time both as a result of their expanded use across settings, populations, and topic areas and also to accommodate scientific developments. For example, the Exploration, Preparation, Implementation, Sustainment (EPIS) framework was initially conceptualized in a more linear form [9], but as a result of its application in the international context, it evolved into a more circular model.

The RE-AIM framework was conceptualized over 20 years ago to address the well-documented failures and delays in the translation of scientific evidence into practice and policy [10]. RE-AIM has been one of the key TMFs that highlights the importance of external validity (e.g., generalizability) in addition to internal validity, with an emphasis on transparency in reporting across all RE-AIM dimensions [7]. It has been one of the most commonly used planning and evaluation frameworks across the fields of public health, behavioral science, and implementation science [7]. There have been over 700 publications that explicitly use RE-AIM for planning, evaluation (most often), or both. RE-AIM has been applied in a wide range of settings, populations, and health issues across diverse clinical, community, and corporate contexts [7,11,12], including policy and environmental change.

RE-AIM dimensions operate and are measured at both the individual level and multiple ecologic levels (most frequently at staff and setting levels in health systems, although it has also often been applied at community and national levels). Its key dimensions are **reach** and **effectiveness** (individual level), **adoption** and **implementation** (staff, setting, system, or policy/other levels), and **maintenance** (both individual and staff/setting/system/policy levels) [7]. Table 1 outlines each RE-AIM dimension with a definition, reporting recommendations, and an example. Reviews indicate that all RE-AIM indicators are not always addressed (e.g., adoption and maintenance are less commonly reported) [13,14], and in recent years, there has been greater emphasis on pragmatic application of the framework a priori with stakeholders to determine which dimensions should be prioritized for improvement, which can be excluded, which measured, and how they should be operationalized [7,15,16]. This evolution is discussed in detail in Glasgow *et al.* [7] and briefly summarized below.

**Table 1.** Clarifications of and reporting on RE-AIM dimension

RE-AIM dimension and definition	Reporting clarifications	Example project
Reach (Individual level): The absolute number, proportion, and representativeness of individuals who are willing to participate in a given initiative, intervention, or program, and reasons why or why not.	Report on both the percentage of individuals who participate based on a valid denominator AND characteristics of individuals who participate compared to nonparticipants. Exclusion criteria and use of qualitative methods to understand reach and/or recruitment are recommended.	DeMarchis *et al.* examined social risk screening by patients and parents in pediatric clinics. Reach quantitatively measured the percentage of participants who actually completed the screening compared to who was approached by site and compared the demographic and background characteristics of participants to nonparticipants [45]. For qualitative examination, interviews with participants, clinicians, and staff explored the motives of those who did and did not participate [46].
Effectiveness (Individual level): The impact of an intervention on important individual outcomes, including potential negative effects, and broader impact including quality of life and economic outcomes; and variability across subgroups (generalizability or heterogeneity of effects).	Report on the primary outcome, measures of broader outcomes (e.g., quality of life), and short-term attrition and differential results by patient characteristics. When possible, measures of robustness across subgroups and use of qualitative methods to understand the outcomes should be reported.	Jauregui *et al.* evaluated the effects of physical activity public health programs in Mexico. Effectiveness outcomes across programs included: weight, BMI, waist circumference, and increased physical activity engagement. Did not include assessment of impact across subgroups. Three of 12 programs conducted qualitative evaluation describing effectiveness [47].
Adoption (multiple setting and staff levels): The absolute number, proportion, and representativeness of settings and intervention agents (people who deliver the program) who are willing to initiate a program, and why. Note, adoption can have many (nested) levels; e.g., staff under a supervisor under a clinic or school, under a system, under a community.	At the setting level (and often at multiple levels relevant), at minimum report on the percentage of settings approached that participated and characteristics of settings participating compared to either nonparticipating settings or some comparable data. At the staff level, report percentage of staff who were invited to participate, percentage excluded, and characteristics of staff participants versus nonparticipating staff.	Kwan *et al.* examined adoption of collaborative care in primary care settings. They measured the decision to adopt by the settings (practices) and the number, percentage, and types of (a) practices and (b) clinicians and staff actually adopting collaborative care. They conducted qualitative interviews to assess clinician reasons for adoption/nonadoption [48].
Implementation (Multiple settings and especially delivery staff level): The fidelity to the various elements of an intervention’s key functions or components, including consistency of delivery as intended and the time and cost of the implementation. Importantly, it also includes adaptations made to interventions and implementation strategies and reasons for the above results.	Not only focused on fidelity, but the adaptations and resources required. Report on the consistency and adherence of intervention delivery, consistency of intervention delivery across settings and/or staff, adaptations made to the intervention and implementation strategies during the study, and costs of the intervention (e.g., time, money). It is especially important to report on the type, timing, and reasons that adaptations are made.	Holtrop *et al.* evaluated implementation of care managers in primary care practice, reporting on implementation fidelity to core components (e.g., multiple sessions over time, use of motivational interviewing) and implementation strategies (e.g., training for practice teams, tickler system for providers). Also reported adaptations (e.g., modification of the electronic health records data capture system, variable training backgrounds for the care managers) and cost to deliver the program [49].
Maintenance (individual and setting levels): At the setting level, the extent to which a program or policy becomes institutionalized or part of the routine organizational practices and policies. At the individual level, maintenance has been defined as the long-term effects of a program on outcomes after a program is completed. The specific time frame for assessment of maintenance or sustainment varies across projects.	Atthe setting level, report if the program is still ongoing at different times after the research funding period, adaptations made to the program post study, and alignment with organizational goals. At the individual level, report on the primary outcome at various points after final intervention time point (recent applications recommend 2 years, modified depending on the issue and other contextual factors), broader health outcomes, long-term attrition, and treatment effect heterogeneity.	Toobert *et al.* evaluated the individual- and setting-level maintenance of a heart disease prevention program on older women with diabetes. Setting-level data included whether the settings could sustain the program over time without added resources and leadership. Also examined were principles to enhance long-term improvements in participants such as follow-up contact, community resources, peer support, and ongoing feedback. At the individual level, they assessed participants’ ability to maintain behavior changes across multiple risk factors at 6–12 months, and then again at 24 months [50].

RE-AIM, reach, effectiveness, adoption, implementation, and maintenance.

A soon to be published research topic in the journal *Frontiers in Public Health*, “Use of the RE-AIM Framework: Translating Research to Practice with Novel Applications and Emerging Directions” provides excellent examples and guidance on the wide range of rapidly expanding applications of RE-AIM for planning, adaptation, and evaluation purposes across diverse settings and populations.

The source RE-AIM publication by Glasgow *et al.* [10] introduced the “RE-AIM evaluation model, which emphasizes the reach and representativeness of both participants and settings” (p. 1322). This is likely the basis for some misconceptions, and especially the repeated classification of RE-AIM as only an evaluation model [17]. Subsequent RE-AIM publications clarified the value of the model for planning, designing for dissemination, and addressing research across the translational science spectrum. As RE-AIM has evolved, it has been integrated into the assessment of the generalizability of findings in the Evidence-Based Cancer Control Programs repository [18] of evidence-based cancer prevention and control interventions and identified as a recommended implementation science model in NIH and foundation funding announcements.

As RE-AIM continues to evolve, it is well positioned to address the realist evaluation question of what intervention components are effective, with which implementation strategies, for whom, in what settings, how and why, and for how long [19]. This contextualized evidence makes RE-AIM practical for replicating or adapting effective interventions in a way that will fit and be feasible for one’s local delivery setting. RE-AIM is less concerned with generating evidence on the question “In general, is a given intervention effective?” and more with impact on different dimensions and outcomes related to population health and the context in which they are and are not effective.

### Understanding and Using RE-AIM: Common Misconceptions and Clarifications

The section below describes and provides guidance regarding common misconceptions of RE-AIM. These misconceptions were identified by polling members of the National RE-AIM Working Group [20], which consists of individuals very active in research using RE-AIM. It includes the authors of this paper, the primary developer of RE-AIM, researchers with the highest number of RE-AIM publications, and those who host the RE-AIM.org website. These individuals are frequently asked to serve on review panels and to review manuscripts related to RE-AIM, as well as to present on RE-AIM at conferences, training programs, and invited lectures. Many of the misconceptions were identified through these processes as well as inquiries to the RE-AIM website. Although a new extensive quantitative review of the frequency of these misconceptions may have added justification, it is unlikely that conclusions of relatively recent systematic reviews of RE-AIM applications [12–14] would have changed. More importantly, a review of the published literature could not include primary sources of many of these misconceptions that have occurred in presentations, manuscript, and grant proposal reviews, training programs, and never-published papers.

Thirteen misconceptions are summarized in Table 2 to illustrate the evolution of the model and clarify the current guidance. Misconceptions identified through the process above were listed, redundancies removed, and very similar issues combined and then organized into broader categorizes. The order of the 13 misconceptions is not prioritized or listed in terms of frequency, but presented under the categories of conceptual, methodological, and implementation issues for ease of understanding. A narrative explanation of each misconception is provided below.

**Table 2.** RE-AIM misconceptions including misunderstanding of the original model, evolution of the model, and the current guidance

Misconception	Correct and current guidance	Potential source of confusion
Conceptual issues (what RE-AIM is and intended to be)		
RE-AIM is exclusively or primarily an evaluation framework	(1) RE-AIM has been used as a process framework to plan implementation and adaptations; (2) PRISM adds a determinants component; and (3) RE-AIM is widely used for evaluation	Misunderstanding of the original intent with some evolution to PRISM
RE-AIM only applies to two levels: individual and organizational	RE-AIM is multilevel although it may be tailored to each project but almost always includes individual, delivery staff, and setting (itself often multilevel)	The original model did not provide examples, but there was never an intent to restrict application to these two levels
RE-AIM (or Expanded RE-AIM/PRISM*) does not include contextual factors, or suggest ways to enhance outcomes	The Expanded RE-AIM/PRISM focuses on contextual factors and addresses ways to enhance RE-AIM outcomes	The model has evolved to PRISM that includes specific contextual factors
RE-AIM does not address (or clearly define/distinguish) longer term sustainment and is restricted to an arbitrary 6-month time frame	Addresses shorter and longer term sustainment (multilevel maintenance) and tailors length of assessment to program	Misunderstanding with the original model stating “at least 6 months following” program completion
RE-AIM does not include costs	Cost is specified as one of the key issues in the Implementation Outcome dimension	Costs are currently reported under Implementation; however, there are costs associated with all dimensions
RE-AIM considers fidelity as the only implementation outcome	Emphasizes both fidelity (consistency) and adaptations	Misunderstanding of original model although adaptation has evolved to be more important
RE-AIM does not account for different phases of implementation and focuses only on postintervention summative effects	Focuses on RE-AIM issues in planning, delivery, evaluation, and sustainment phases	Original model always included consideration of phases, but had not been explicitly stated; increased emphasis on use before, during, and after implementation
Methodological issues (how RE-AIM and RE-AIM dimensions are used)		
RE-AIM uses only quantitative data	Includes measures and guidance for both qualitative and quantitative assessment	Misinterpretation of the original model; qualitative has always been recommended, however, increased emphasis and guidance for use more recently
RE-AIM is static – meaning it does not address adaptations and is not used iteratively	Used for iterative assessment and guiding adaptations	Has been used informally to guide iterations, but more recently an explicit protocol for iterative use is available
RE-AIM insists on using all dimensions in every project and that all dimensions are equally important in every application	Pragmatic use emphasizes considering all dimensions but tailoring (a) which are assessed; (b) which are the intervention focus; and (c) how outcomes are weighted to be tailored to each project	Misinterpretation of the original model and evolving emphasis on pragmatic use
Use of the model issues (clarity of ways to use RE-AIM)		
RE-AIM constructs are difficult to distinguish	Specific definitions, clarifications, and examples are provided of differences among dimensions	Clarifications of model dimensions are increasingly available
RE-AIM only works for research or in large, well-funded studies	Scope and depth of use of RE-AIM for planning, iteration, and evaluation can be tailored pragmatically to fit each project	Always available for any type of project, however, more and better examples of diverse uses are now more available
Use of RE-AIM precludes use of other implementation science frameworks in the same project	RE-AIM can be combined with other TMFs** and examples of integration are provided	Use with other frameworks is increasingly encouraged

RE-AIM is reach, effectiveness, adoption, implementation, and maintenance.

* PRISM is the practical, robust, implementation, and sustainability model.

** TMF refers to theories, models, and frameworks.

### Conceptual Issues

#### RE-AIM is exclusively or primarily an evaluation framework

RE-AIM has always been designed to help translate research into practice [7,10,15,21]. Early conceptualization of RE-AIM focused on the application of a comprehensive evaluation framework targeting multilevel interventions [10]. While evaluation continues to be a focus, as early as 2005, Klesges *et al.* [22] explicitly focused on RE-AIM as a tool to design programs, and Kessler *et al.* (2013) [23] explicated full use of RE-AIM, synthesizing program planning, implementation, evaluation, and reporting with a focus on external validity. Thus, while evaluation was an early focus, RE-AIM emphasizes translation of research to practice, designing for dissemination, and the importance of considering context in planning and design, in addition to evaluation.

#### RE-AIM only applies at two levels: Individual and organizational

RE-AIM applies to many socioecologic levels, and understanding different levels is a common source of confusion. This may have resulted from early applications only providing examples at the individual (e.g., patient, employee, student) and organizational (e.g., clinic, workplace, school) levels and the explicit use of the R and E domains at the patient level and the A, I, and M domains at the organization level. However, there was never an intent to restrict application to these two levels. More recently, RE-AIM domains are applied at three or more levels [24].

The dimension within RE-AIM where multilevel application is most evident is the expansion of “adoption,” i.e., the settings where interventions are delivered, to include multiple levels of delivery agents and contexts, in addition to organizations. Given that human behavior takes place in, and is influenced by, multilevel contexts [25], and that exposure to an intervention across multi-level “settings” (e.g., policy, neighborhood, and organizations) may be necessary to maximize reach, collective impact, and sustained effectiveness [24], potential adopters could be policy makers, agencies, councils, urban planners, municipal departments, or community groups.

This broadened definition admittedly makes measurement of adoption more challenging as there are often multiple levels of nesting (e.g., clinics within health systems within regions). If the intervention is a policy change (e.g., national policy requiring nutrition labeling of packaged food), or an environmental change (e.g., open space improvement project to encourage physical activity), implementation strategies and their relevant metrics may include participation and engagement of decision-makers, supportive agencies, and enforcers. Specifying which key stakeholders are relevant during planning, approval, implementation, and sustainment phases, and which agency or individuals will be responsible for maintaining (or enforcing) the program or policy is key. Strategies for defining and measuring adoption should be tailored to one’s specific application.

#### RE-AIM does not include “determinants,” contextual factors, or suggest ways to enhance outcomes

**Fig. 1.**
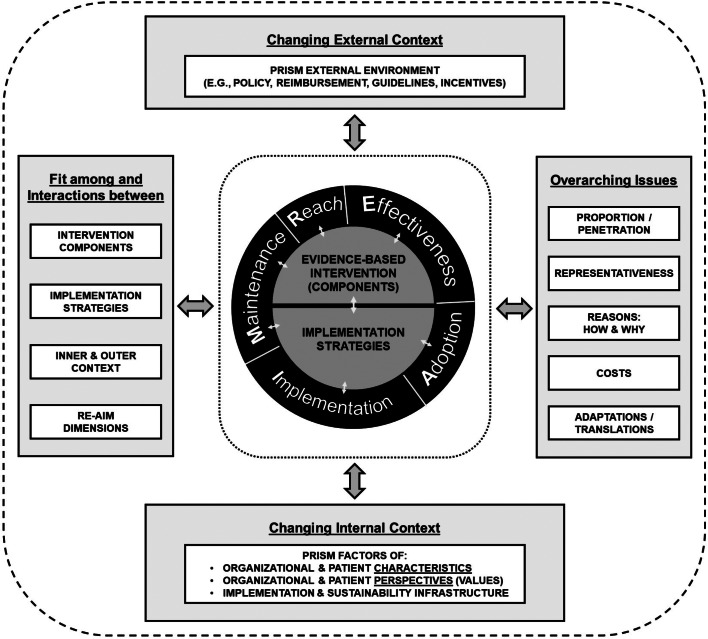
Pragmatic Robust Implementation and Sustainability Model (PRISM).

The original publication on and early research using RE-AIM did not include determinants. However, RE-AIM was expanded several years ago into PRISM [7,26]. The Practical Robust Implementation and Sustainability Model or PRISM includes RE-AIM outcomes (center section of Fig. 1) [26] and explicitly identifies key contextual factors related to these outcomes (see outer sections of Fig. 1). PRISM was developed in 2008 to address context and to provide a practical and actionable guide to researchers and practitioners for program planning, development, evaluation, and sustainment. It includes RE-AIM outcomes and also draws upon and integrates key concepts from Diffusion of Innovations [27], the Chronic Care Model [28], and the Institute for Healthcare Improvement (IHI) change model [29].

As shown in Fig. 1, PRISM (seewww.re-aim.org) identifies key “determinants” factors such as policies, “implementation and sustainability infrastructure,” and multilevel organizational perspectives [30]. It has been successfully used to guide planning, mid-course adjustments to implementation, and evaluation of interventions across a wide range of settings and topic areas [16], with the original PRISM publication being cited by 160 different papers. The broad applicability of PRISM makes it a useful tool for conceptualizing context [7,26,30]. Feldstein and Glasgow and the RE-AIM website provide reflection questions for each factor for planning [26].

Depending on the study and/or needs, *we recommend that researchers and practitioners who plan to use RE-AIM consider the use of PRISM*. While not all studies or programs will decide to comprehensively evaluate or address context, PRISM can provide a pragmatic, feasible, and robust way to consider important contextual factors. There are new sections of the re-aim.org website that clarify these issues as well as how PRISM relates to RE-AIM.

#### RE-AIM does not address (or clearly define/distinguish) longer term sustainment and is restricted to an arbitrary 6-month time frame

This misconception likely came from the statement in the original RE-AIM publication that maintenance was defined as “*at least* 6 months following program completion” (emphasis added). This was never intended to mean that maintenance should only be assessed at or evaluation stopped at 6 months; there are numerous publications assessing maintenance at much longer intervals [31]. RE-AIM can and should include assessment of long-term sustainment intentions and plans, even if funding limitations preclude assessment at distal time points [7]. Historically, RE-AIM is one of the first frameworks that explicitly called attention to issues of sustainability or maintenance at setting levels. Although systematic reviews of RE-AIM application have found that “maintenance” data are often not reported [14], there has been growing recognition of the importance of understanding of longer term sustainment in a dynamic context [32,33]. A recent extension of RE-AIM addresses this gap and advances research by explicitly conceptualizing and measuring maintenance/sustainability as longer term (i.e., ideally at least 1–2 years post initial implementation and over time) and dynamic in nature [34].

The conceptualization of dynamic sustainability [33] associated with RE-AIM includes consideration of (1) iterative application of RE-AIM assessments to address sustainability throughout the life cycle of an intervention; (2) guidance for operationalizing dynamic sustainability and the “evolvability” of interventions, including continued delivery of the original intervention functions and implementation strategies, adaptations, and potential de-implementation to produce sustained and equitable health outcomes; and (3) continued and explicit consideration of costs and equity as driving forces impacting sustainability across RE-AIM dimensions [34].

#### RE-AIM does not include costs

Cost and economic analyses are central issues in dissemination and implementation in any setting. Although cost has been explicitly included in RE-AIM since at least 2013, it is one of the least frequently reported elements in research reports, while often one of the primary concerns of potential adopters and decision makers [13]. When costs are reported, this is often done inconsistently, making comparisons difficult [35]. Understanding cost is challenging because it varies depending on the complexity of the intervention(s), the implementation strategies used, and the settings for delivery.

Currently, in RE-AIM cost is most explicitly considered and reported under Implementation, and has been for many years [23]. More recently, some RE-AIM applications have included a focus on cost to obtain desired outcomes across all five framework dimensions [23]. Rhodes *et al.* [36] identified and illustrated pragmatic processes and measures that capture cost and resource requirements from the perspectives of individual participants, intervention agents, and settings. Such evaluations from a variety of multilevel stakeholder perspectives are needed to enhance research translation into practice [35].

#### RE-AIM considers fidelity as the only implementation outcome measure

In the original RE-AIM paper, implementation referred to the extent to which a program was delivered as intended (fidelity) [10]. The implementation dimension has evolved dramatically over the last 20 years and now has the most components of any RE-AIM dimension. Currently, implementation evaluation criteria include fidelity to delivering the intervention, consistency of implementation across sites/setting, adaptations made to the intervention or implementation strategies, and, as discussed above, costs [7,23]. Implementation seeks to understand how an intervention is integrated into diverse settings and populations, as well as how it is delivered and modified over time and across settings. The importance of identifying, tracking, and understanding the need for adaptation is central to implementation. Moreover, this dimension has benefitted greatly from expansion and incorporation of qualitative methods, which allow for a deeper exploration and understanding of not only what the adaptations were, but who made them, why they were made, and what the results were [19,30,37,38].

#### RE-AIM does not account for different phases of implementation

Some frameworks [9] explicitly define phases of implementation, and a criticism of RE-AIM is that it does not. Actually, PRISM has been used to address progress on and priority across different RE-AIM dimensions at different points in the implementation process (planning, early and mid-implementation, sustainment) [16]. There is an inherent temporal order in RE-AIM such that a program has to be adopted before it can be implemented, it needs to be adopted, reach participants, and be implemented to be effective, etc. Pairing RE-AIM dimensions, including important questions implementation teams can ask themselves *before*, *during*, and *after* the implementation process, with a contextual determinants framework, such as PRISM [26] or the Consolidated Framework for Implementation Research (CFIR) [39,40], allows selection of the most relevant constructs at different points in time. Implementation teams can then use these constructs to help guide targeted implementation strategies that will ensure maximum reach, effectiveness, implementation fidelity, and maintenance.

### Methods Issues

#### RE-AIM uses only quantitative data

Definitions of RE-AIM constructs have always had a quantitative component; for instance, reach is defined as the number and percent of eligible recipients who actually participate in an intervention and the representativeness of those participants. Yet, reach also includes the type of eligible recipients who participate (including equity in participation and access) and the reasons for participation or nonparticipation. While “type” and “reasons” can be operationalized as quantifiable measures (e.g., participant demographics, frequencies of people citing transportation or cost barriers to participation), there is important nuance and depth to the *who, what, why,* and *how* of reach that numbers may not convey. The same is true for the other RE-AIM dimensions. Qualitative and mixed methods are valuable tools for gaining deep insight into questions of who, what, how, and why people participate in and gain benefit from an intervention [37] and have been explicitly recommended in multiple RE-AIM reviews [14,23]. Holtrop *et al.* (2018) recently provided a thorough synthesis and guide to qualitative applications of RE-AIM [37].

#### RE-AIM is static (meaning not used iteratively)

In addition to RE-AIM’s use during planning, implementation, and sustainment phases of a program as mentioned earlier, RE-AIM can structure the assessment of progress and provide guidance for mid-course adjustments and adaptations by assessing the impact on all dimensions at a given point in time [12]. Although RE-AIM has been used informally to guide iterations for many years [41,42], more recently, Glasgow *et al.* (2020) developed a specific protocol for the iterative use of RE-AIM and described key steps and application of this methodology across five health system studies in the VA [16]. This methodology was found to be feasible and led to reflective conversations and adaptation planning within project teams.

#### RE-AIM requires the use of all dimensions, with all dimensions considered equally important

This misconception is likely based on an over reliance on the original RE-AIM source article that highlighted the importance of all five RE-AIM dimensions and proposed that the potential public health impact was the product of results on all dimensions [10]. Reviews of the RE-AIM literature have consistently found that usually not all RE-AIM dimensions are reported within a single study [14]. This and practical considerations precipitated a more explicit pragmatic approach to applying RE-AIM that relies on stakeholder priorities regarding the relative importance of and need to gather information across the RE-AIM dimensions [15].

Not all RE-AIM dimensions need to be included in every application, but (a) consideration should be given to all dimensions; (b) *a priori* decisions made about priorities across dimensions and reasons provided for these decisions; and (c) transparent reporting conducted on the dimensions that are prioritized for evaluation or enhancement [15]. This pragmatic approach encourages the use of all dimensions during planning, conducting evaluation based on dimensions important for decision-making, and providing contextual information on dimensions that are not directly targeted.

### Implementation of RE-AIM

#### RE-AIM constructs are difficult to distinguish

New users of RE-AIM frequently conclude that reach and adoption are the same thing; that adoption and implementation overlap; or that it is unimportant or redundant to consider both. Concerns that RE-AIM constructs are not distinct or have what has been described as “fuzzy boundaries” [11] have been addressed through education and guidance on RE-AIM measures and application. While RE-AIM has face validity and is intuitive for many people, it does have nuances and requires study to learn and apply appropriately.

Reach is measured at the level of the individual – the intended beneficiary of a program (e.g., a patient or employee). Adoption is measured at one or more levels of the organizational or community setting that delivers the program and the “staff” or delivery agents within those settings (e.g., a healthcare provider or educator decides to offer or be trained in a program) [11]. Similarly, effectiveness refers to outcomes at the recipient level (e.g., increased physical activity, weight loss), while implementation refers to program delivery outcomes at the service provider level (e.g., the interventionist consistently follows the intervention protocol, a healthcare provider consistently screens patients).

#### RE-AIM only works for large-scale research or in large, well-funded evaluations

The value of RE-AIM for nonresearch application was summarized in a recent report on use of RE-AIM in the “real world” – i.e., use in clinical and community settings for planning, evaluating, and improving programs, products, or services intended for improving health in a particular population or setting (not to create generalizable knowledge) [11]. Many successful projects described use of RE-AIM with low budgets or even no budget. Data source, timeline, and scope and scale of a project all influence feasibility, timeline, and resource requirements. Picking one or two high-value metrics or qualitative questions for a RE-AIM-based evaluation and focusing on readily available data sources can help to keep an evaluation within scope. A special issue in *Frontiers of Public Health* in 2015 featured reports from a large number of organizations that—with very little funding—successfully applied RE-AIM to assess physical activity and achieve falls reduction among older adults [43].

#### Use of RE-AIM precludes use of other implementation science frameworks

Use of RE-AIM in combination with other frameworks is common [39] and encouraged [7], assuming that the models are compatible. Combining frameworks that yield complementary information (e.g., process, explanatory frameworks, TMFs from other disciplines) enhances our ability to understand why and how implementation succeeded or failed [39]. When multiple frameworks are used, it is important to provide a theoretical rationale for how the selected frameworks supplement each other [17]. An example of the use of multiple frameworks is Rosen *et al.* [44] who used the EPIS framework, along with CFIR to assess what emerged as a key adoption determinant (client needs and resources), and RE-AIM to assess relevant implementation outcomes (reach and effectiveness). Multiple examples of integrating RE-AIM with other TMFs are provided at www.re-aim.org.

### Resources and Current Directions for RE-AIM

We have taken several steps and developed the resources summarized below to address the above misconceptions. We also briefly note ongoing activities that we hope will also help to provide additional clarification and guidance.

### RE-AIM Website Revisions, Expansion, and Other Resources

Guidance on appropriate use of the RE-AIM (and other TMFs), beyond peer-reviewed publications, is needed to address these misconceptions. In recognition of this need, members of the National RE-AIM Working Group [20] developed a website in 2004 (www.re-aim.org) to assist with the application of RE-AIM. The website provides definitions, calculators, checklists, interview guides, and a continuously updated searchable bibliography. The website has expanded to include blog posts, slide decks (one specifically on misconceptions), and a webinar series. Answers to questions about how to apply the framework, FAQs (http://www.re-aim.org/about/frequently-asked-questions/), and evidence for RE-AIM have been added or expanded.

Of particular relevance for readers of this article, we have recently (a) added new summary slides on RE-AIM that address many of the issues above (http://www.re-aim.org/recommended-re-aim-slides/) and (b) created a new summary figure that illustrates the PRISM/RE-AIM integration and highlights several crosscutting issues that should provide clarification on some of the misconceptions above (Fig. 1) [7]. The website is updated monthly, and a new section provides more examples and guidance for applying RE-AIM, including a checklist for users to self-evaluate their use of RE-AIM. Finally, members of the current National Working Group on the RE-AIM Planning and Evaluation Framework published a recent paper describing the use of this website platform to disseminate a number of resources and tools [20].

### Non-Researcher Use of RE-AIM

We have addressed the misconception that RE-AIM only works for controlled research or large, well-funded studies. However, applying RE-AIM in nonresearch settings often requires guidance and direction for optimal utilization by those not familiar with RE-AIM [11]. A basic understanding of RE-AIM concepts and methodologies can be obtained from pragmatic tools on the website and key publications demonstrating successful application of RE-AIM model in community or clinical settings [11,12]. For more complex issues, we also recommend more interactive consultations with an experienced RE-AIM user or the National Working Group.

### Current Directions

Over time, several core aspects and features of RE-AIM have remained the same, but others have substantially evolved [7]. The key principles and dimensions remain, and there is still an emphasis on actual or estimated public health impact and generalizability of findings. The changes to RE-AIM have been in response to growing knowledge in implementation science and practice and include (a) refinement of the operationalization and some expansion of the key dimensions; (b) incorporation of context, adaptations, health equity, and costs; and (c) guidance on applying qualitative approaches.

We expect that as our methodological approaches and measurement practices advance, RE-AIM/PRISM will continue evolving as recommended by Kislov *et al.* [3] Our key current emphases are summarized in Table 3 and include more in-depth assessment of context as part of PRISM. We are developing and testing standardized survey measures of RE-AIM dimensions, as well as guidance for iterative application of RE-AIM to inform adaptations [16]. Other current directions include application of expanded RE-AIM/PRISM to address the interrelated issues of cost, value, and sustainability [34], and explication of how representativeness components of RE-AIM helps address health equity. We are also exploring the integration of RE-AIM with participatory systems dynamic modeling and other stakeholder engagement methods.

**Table 3.** Current directions and resources under development for the expanded RE-AIM/PRISM framework

Focus or direction	Issue description and current activities
Context	Enhancing, refining, and reporting on PRISM contextual factors – new website sections and interactive guidance tool
Measures and norms	Developing, validating, and providing standard survey questions for RE-AIM dimensions
Rapid and iterative use	Replicating, expanding, and investigating ways to optimize repeated serial use of RE-AIM for during implementation assessment and guiding adaptations [16]
Cost, value, and sustainability	Expanding cost components of RE-AIM to include not only time and financial costs but also stakeholder perceptions of burden and value and their relationship to sustainment [36]
Health equity	Specifying RE-AIM perspectives (especially factors related to context and representativeness) on and contributions to health equity issues [34]
Modeling and stakeholder engagement	Integrating RE-AIM with participatory systems dynamic modeling and other stakeholder engagement methods [42]
Guidance and training	Enhancing guidance and examples provided regarding application of RE-AIM, including trainings, tools, guides, and examples – available on website
Communication activities	Enhancing, updating, and expanding the RE-AIM website and developing other communication channels to provide more resources for (a) different types of users and (b) different phases of research/evaluation – including @reaim tweets and RE-AIM list serve for announcements

PRISM, Pragmatic Robust Implementation and Sustainability Model; RE-AIM, reach, effectiveness, adoption, implementation, and maintenance.

## Conclusions

TMFs have contributed substantially to implementation science [1,2]. They evolve over time to address limitations and applications to new contexts and to improve their usefulness [3]. This evolution can create confusion about the possible, correct, and best uses of the TMF. In light of these issues, we make the following recommendations to reduce confusion and misunderstanding about the use of a TMF while also acknowledging that acting on such recommendations might not always be feasible.

1. With each major modification or update of a TMF, *developers* could produce a scholarly publication and an archived webinar that explicitly describes the modification, the problem it is addressing, and current guidance. It may help to include explicit language such as “An adaptation to TMF XX” to make it clear this is a revision, or an expanded version of the TMF. If the revision is not produced by the originators of the TMF, the developers should be consulted, and the publication should state whether they agree with the change.
2. Although sometimes challenging, *reviewers* who are summarizing or categorizing a TMF should check the recent literature and major users of that TMF to make sure descriptions of it are accurate.
3. Potential *users* of a TMF should complete a review, especially looking for recent updates, and explicitly search for adaptations to the TMF. In particular, they should check the website on the TMF, if one exists. Users should cite more recent uses of the TMF; not only the original publication or an earlier review. Finally, it is important for TMF users to carefully articulate why and how they are using a TMF, which may help alleviate some issues of mischaracterization.
4. *Hosts of TMF websites* should summarize improvements or revisions to the TMF in a prominent location and explicitly identify common misconceptions and what to do about them.

As Kislov and colleagues [3] have articulated, TMFs should change and adapt over time so that they do not become ossified. Table 3 outlines ways in which RE-AIM is currently evolving and resources that are being developed. When TMFs do change and adapt, it is important that researchers and users keep up with such changes. Although it is impossible to completely prevent such occurrences, we hope this discussion and our recommended actions will minimize TMF misapplication. We welcome dialogue about actions that can be taken by TMF developers, reviewers, and users.
